# IgE Antibody Function and Mast Cell Activation to Egg in Children Undergoing Baked and Loosely Cooked Egg Challenges

**DOI:** 10.1111/all.70368

**Published:** 2026-04-29

**Authors:** Vikki Houghton, Vasco Claro, Nur Romli, Zainab Jama, Grammatiki Antoneria, Matthew Kwok, Alexandra F. Santos

**Affiliations:** ^1^ Peter Gorer Department of Immunobiology, School of Immunology and Microbial Sciences King's College London London UK; ^2^ Department of Basic and Clinical Neuroscience, Institute of Psychiatry, Psychology and Neuroscience King's College London London UK; ^3^ Department of Women and Children's Health (Pediatric Allergy), School of Life Course Sciences, Faculty of Life Sciences and Medicine King's College London London UK; ^4^ KCL School of Bioscience Education, Faculty of Life Sciences and Medicine King's College London London UK; ^5^ Children's Allergy Service Evelina London Children's Hospital, Guy's and St Thomas' Hospital London UK

**Keywords:** basophil activation test, egg allergy, food allergy, IgE, mast cell activation test

## Abstract

**Background:**

An accurate diagnosis of egg allergy is extremely important. Understanding egg‐specific antibody function can inform future therapeutic strategies. We aimed to assess the diagnostic performance of the mast cell activation test (MAT) to egg and how IgE characteristics and IgG4 modulate mast cell response to egg allergens.

**Methods:**

BAT2 egg study participants with complete clinical outcomes and samples available for MAT were grouped based on oral food challenges (OFC) to baked egg (BE) and loosely cooked egg (LCE). Raw egg allergy was not assessed. IgE levels, specific activity, diversity, avidity for egg allergens and ratios of egg‐specific IgG4/IgE were compared between baked egg allergic (BEA) and baked egg tolerant (BET) children and related to MAT and basophil activation test (BAT) to egg.

**Results:**

BAT2 egg study participants studied (*n* = 133) included 58 BEA and 75 BET (16 LCE allergic and 59 LCE tolerant). MAT to egg showed an area under the ROC curve (95% CI) of 0.749 (0.663; 0.835) for BEA and 0.704 (0.616; 0.793) for LCE allergy. Sensitivity/specificity of MAT's optimal cut‐off was 52%/89% for BE allergy and 70%/66% for LCE allergy. Diagnostic accuracy of MAT was 68% for both forms of egg allergy, which was lower than that of other tests. MAT was dependent on the presence of allergen‐sIgE and had superior diagnostic accuracy to sIgE only when levels sIgE ≥ 1 KU/L. BAT and MAT results were moderately correlated (*R*s = 0.431, *p* < 0.001). IgE levels, specific activity and avidity for egg allergens were directly correlated, and egg‐specific IgG4/IgE ratios were inversely correlated with MAT to egg.

**Conclusion:**

MAT distinguishes BEA from BET children and is a suitable tool to test IgE function and the interference of IgG4 in mast cell response to egg allergens.

## Graphical abstract

MAT is dependent on the presence of allergen‐specific IgE and can provide false‐negative results in allergic patients with low allergen‐specific IgE levels. MAT and BAT are moderately correlated. MAT can be used as a surrogate of BAT, when fresh blood is not available or in the case of patients with non‐releaser basophils. MAT is directly correlated with allergen‐specific IgE levels, specific activity and avidity and inversely correlated with egg‐specific IgG4/IgE ratios indicating that functional characteristics of IgE are relevant to and that allergen‐sIgG4 can interfere with IgE‐mediated mast cell activation following allergen exposure. BAT, basophil activation test; DBPCFC, double‐blind, placebo‐controlled food challenge; Ig, immunoglobulin; KU/L, kilo units per litre; LAD2, human mast cell line; MAT, mast cell activation test; NCT, national clinical trial.

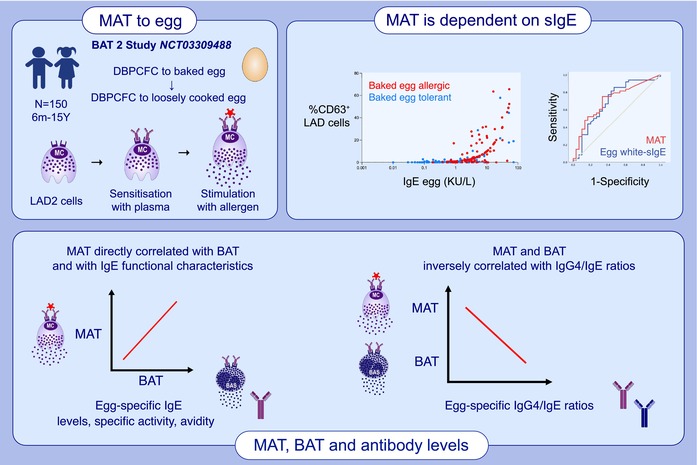

## 1 Introduction

Egg allergy is one of the most common food allergies in childhood and a major cause of anaphylaxis in infants [1, 2]. It resolves spontaneously in most children by school age [3, 4, 5, 6]; however, in some affected individuals, it persists throughout life [2, 7]. Being a ubiquitous allergen, accidental reactions to egg are common. The fear of encountering the allergens can cause anxiety and plays a large part in the burden of food allergy on the lives of allergic patients and their families. Given the negative impact of egg allergy on patients and society, diagnosing egg allergy with precision is fundamentally important. Learning from spontaneous resolution could pave the way for new treatments; however, the immune mechanisms underlying this phenomenon are not completely understood.

Current diagnostic tests, such as skin prick test (SPT) and specific IgE (sIgE), are not entirely accurate for egg allergy, even when the clinical history is considered [8]. Specific IgE to egg components does not provide additional information compared to egg or egg white‐sIgE [9, 10]. We have previously demonstrated that the basophil activation test (BAT) can improve the diagnosis of egg allergy and reduce the need for risky, time‐consuming oral food challenges (OFC) [11]. However, BAT requires fresh blood and processing soon after blood collection. The mast cell activation test (MAT) has the advantage of using only serum or plasma, which can be stored frozen for long periods of time. However, because MAT requires transference of patients' antibodies to heterologous cells that may have a different density of IgE receptors expressed on their surface, MAT constitutes a more artificial system that is removed further away from the potentially allergic patient and their clinical phenotype compared to BAT.

As it relies on passive sensitisation and the cells are a constant, MAT is suitable to assess the ability of IgE from different patients to mediate mast cell activation and degranulation following allergen stimulation [12]. Similarly, it is well suited to assess the interference of blocking antibodies, such as IgG4, on IgE‐mediated mast cell activation [13]. We have previously demonstrated the utility of the MAT that uses LAD2 cells in the diagnosis of peanut allergy [13, 14] and used as a tool to assess the ability of IgE antibodies with different characteristics to induce mast cell activation following peanut allergen stimulation [12, 15, 16]. In this study, we used samples from participants in the BAT2 egg study [11, 17] to assess the diagnostic accuracy of the MAT to support the diagnosis of egg allergy and to reflect the function of IgE and IgG4 interference in the IgE‐allergen interaction.

## 2 Methods

### 2.1 Study Design

**FIGURE 1:**
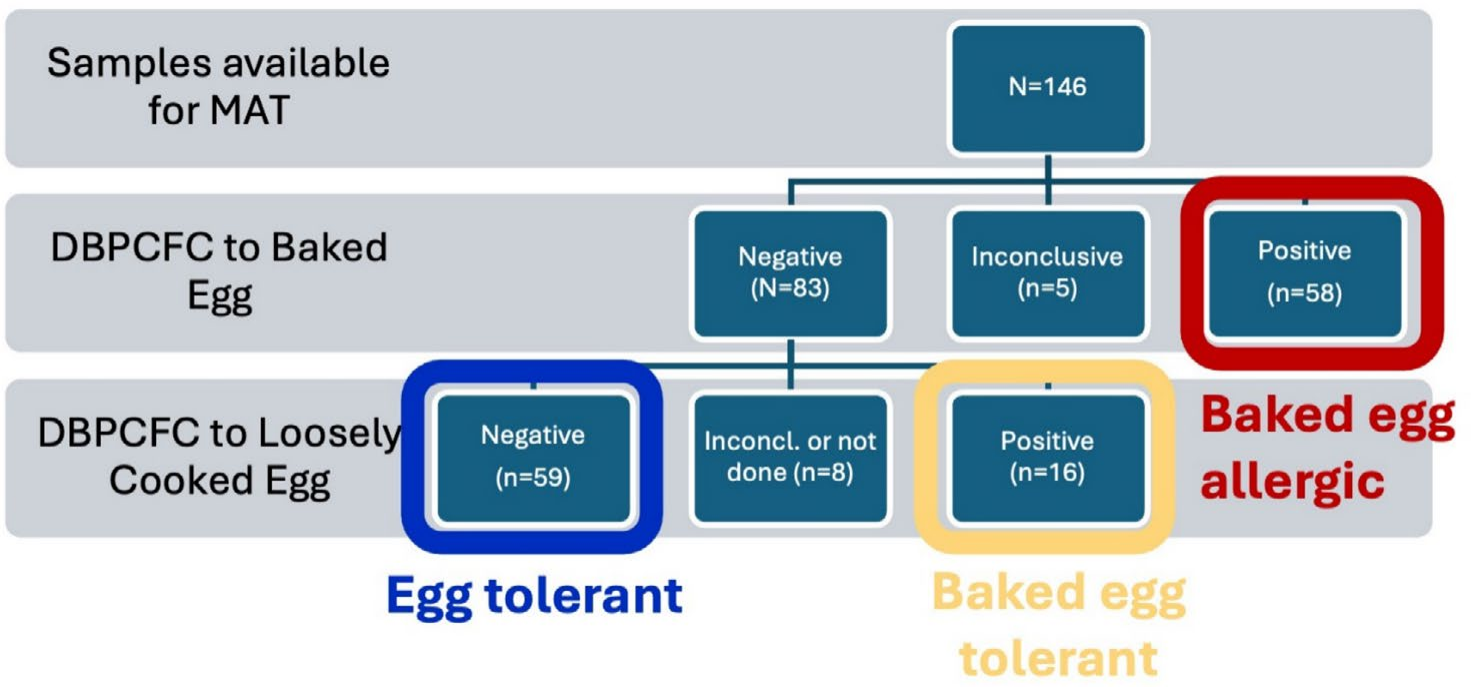
Flow of BAT2 participants in the study according to availability of samples to test on the mast cell activation test and clinical outcomes following double‐blind placebo‐controlled food challenges (*n* = 146). Thirteen participants had inconclusive results or did not attend their oral food challenge.

Participants in the BAT2 egg study (registered in clinicaltrials.gov with NCT03309488) with available plasma samples for testing on MAT were included in this study. The BAT2 study was approved by the London Westminster Research Ethics Committee (reference number 17/LO/0296). Adults with parental responsibility signed informed consent prior to study procedures. As previously reported [11, 17], BAT2 study participants had clinical assessment, skin prick test (SPT) and blood collected on the day of their baked egg OFC for serology, BAT and MAT. In case of no reaction, they attended a second visit when they had an OFC to loosely cooked egg. Patients were grouped according to the outcome of both baked egg and loosely cooked egg OFC in one of three clinical phenotypes: baked egg allergic, baked egg tolerant and tolerant to both forms of egg (Figure 1). Clinical parameters such as eczema, asthma and allergic rhinitis were defined based on physician‐diagnosis—eczema included a history of or current eczema, whereas respiratory allergies were defined based on current disease. Asthma included recurrent wheeze.

### 2.2 Clinical Procedures

SPT was performed using a metal lancet, egg white extract (ALK Abello, Madrid, Spain), 10 mg/mL histamine dihydrochloride as positive control and 50% glycerol and 50% buffered saline as negative control. Skin weal diameter was recorded after 15 min as the mean of two perpendicular diameters including the longest one. Blood was collected for serology, BAT and MAT. All children had OFC; infants < 12 months of age had an open incremental OFC and all other participants had a one‐day DBPCFC. In case of reaction to placebo, a 2‐day DBPCFC was done. All negative DBPCFCs were followed by an age‐appropriate final open dose. Dosing regimens were adapted for different age groups, as previously published [11, 17] and shown in Tables S1 and S2 [11]. Both baked egg and loosely cooked egg doses were prepared by the clinical research staff according to a standardised food preparation protocol (see Supporting Information S1 for details). Raw egg allergy was not assessed, thus, patients tolerating LCE may have still reacted to raw egg. The outcome of the OFC was determined according to the Practall guidelines [18].

### 2.3 IgE and IgG4 Testing

The levels of total IgE and specific IgE and IgG4 to egg, egg white, ovomucoid (Gal d 1) and ovalbumin (Gal d 2) were quantified in patients' serum using the ImmunoCAP assay (Thermo Fisher, Uppsala, Sweden), and analysed using Phadia 100 (Thermo Fisher, Uppsala, Sweden) according to the manufacturer's instructions.

### 2.4 IgE Specific Activity

Egg white‐sIgE specific activity (SA) was determined by calculating the ratio of egg white‐sIgE to total IgE levels, measured using ImmunoCAP assays as detailed above and expressed as a percentage, using the following formula:

$$\frac{\text{sIgE to}\;\text{egg}\;\text{white}}{\text{Total}\;\text{IgE}}\times 100$$

### 2.5 IgE Diversity

For each patient, the results of sIgE to ovomucoid and ovalbumin were transformed into diversity index, H, using the following formula [12]:

$$H=-\sum_{i=1}^{S}p_{i}\;\ln\;p_{i.}$$

where *H* = diversity index; *i* = (1, … *S*), *S* = total number of allergens measured, *p*_*i*_ = the proportion (relative abundance) of allergen, *i*, in total amount of allergen specific IgE levels.

### 2.6 IgE Avidity

Avidity measurements require a 1:1 dilution with either PBS or a chaotropic agent; therefore, samples with a minimum of egg‐sIgE level of 1kU_A_/L or more were selected. The chaotropic agent used was sodium thiocyanate (NaSCN). Patients' plasma were incubated with either PBS or NaSCN of the optimum concentration for 20 min at room temperature. The samples were then run on ImmunoCAP to check for the level of egg‐sIgE. The avidity of patients' sIgE was described as the proportion of IgE binding decrease and was calculated using the following formula [12]:

$$100-\frac{\text{sIgE level with NaSCN}}{\text{sIgE level with}\;\mathrm{PBS}}\times 100$$

To determine the optimal concentration of NaSCN used for egg‐sIgE avidity measurements, three samples with low, medium and high levels of egg‐sIgE were tested with increasing concentrations of NaSCN from 0 to 6 M, diluted 1:1 (Figure S1). The optimal concentration of 2 M NaSCN was used for subsequent avidity measurements.

### 2.7 Basophil Activation Test

The BAT was performed as previously reported [11, 17]. Whole blood was incubated with an equal volume of stimulant, including RPMI (GIBCO, UK), egg extract (ALK‐Abello, Madrid, Spain) or baked egg (Sigma‐Aldrich, UK), anti‐IgE (Sigma, UK) or formyl‐methionyl‐leucylphenylalanine (Sigma‐Aldrich, UK), for 30 min at 37°C in an incubator. Cells were stained with CD123‐FITC, CD203c‐PE, HLA‐DR‐PerCPCy5.5 and CD63‐APC (Biolegend, San Diego, CA, USA) for 30 min at 4°C and lysed with Pharmlyse (BD Biosciences, San Diego, CA) for 15 min at room temperature. After a final wash, cells were analysed by Flow Cytometry on Fortessa (BD Biosciences, San Diego, CA) and analysed with FlowJo (version 10.7.1).

### 2.8 Mast Cell Activation Test

MAT was performed as in previous studies [13, 14, 19]. LAD2 cells (Laboratory of Allergic Diseases, National Institute of Health, Bethesda, Md) [20] were cultured in the presence of 10 ng/mL recombinant human Interleukin 4 (IL‐4) at 37°C for 5 days. The cells were then sensitised using patients' plasma in a 1:4 dilution and incubated at 37°C overnight. Plasma samples were stored in 1 mL volumes and at least 100 μL was used per MAT. Subsequently, the cells were resuspended in HEPES buffer to obtain 2 × 10^5^ cells/mL. Using a 96 well U bottom plate, 10 μL of stimulant (1 μg/mL aIgE, 1 μM ionomycin, 1000 ng/mL of egg extract or medium) was added to the cell suspension and incubated at 37°C for 30 min. Each stimulation condition was performed in a single replicate. To stop the reaction, 20 mM of EDTA was added into each well and the plate was then placed on ice. The cells were stained by adding CD63‐APC (Biolegend, San Diego, CA) and incubated at 4°C in the dark for 30 min. Viability Dye eFlour 450 (eBioscience, San Diego, CA) was added into each well and incubated at 4°C in the dark for 30 min. After a wash, the samples were analysed on CytoFLEX flow cytometer and the data using FlowJo software (version 10.7.1). Mast cells were gated as (Figure S2). After data analyses, background subtraction was applied so that all stimulation conditions (extract and anti‐IgE) were corrected for the negative control levels. Operators were blinded to patient phenotype in all cases.

### 2.9 Statistical Analyses

Statistical analyses were performed using GraphPad Prism 9.5.1 (GraphPad Software, San Diego, CA) and SPSS 27.0 (IBM SPSS Statistics New York). Kolmogorov–Smirnov test was done to determine the normality of the data distribution. Mann–Whitney U test and Kruskall Wallis test were used for comparison of 2 or more independent groups, respectively. Wilcoxon test was used for pairwise comparisons. Diagnostic performance was measured using ROC curve analyses. Optimal cut‐offs were based on the Youden index. The association of each IgE characteristic and MAT or BAT was analysed using Spearman's correlation. The data were considered statistically significant when *p* < 0.05.

## 3 Results

### 3.1 Study Population

In this study, 146 participants in the BAT2 egg study who had plasma samples available were tested on the MAT to baked egg and to egg extract. Depending on the outcome of the DBPCFC to baked egg and to loosely cooked egg, the 133 patients with a complete clinical outcome were grouped into baked egg allergic (*n* = 58), baked egg tolerant (*n* = 16) and egg tolerant (*n* = 59) – Figure 1. There were 13 patients who did not have an outcome for OFC to baked egg (*n* = 5) or loosely cooked egg (*n* = 8). Their characteristics are presented in Table S3.

Table S4 summarises the demographic and clinical characteristics of this cohort (*n* = 133). Most patients were either allergic (44%) or tolerant (44%) to all forms of egg, and a minority (12%) had the intermediate phenotype of tolerating baked egg but still reacting to loosely cooked egg. There were no statistically significant differences in demographic or clinical characteristics between the 3 groups, except for asthma being more prevalent in the group reactive to all forms of egg. The results of SPT to egg, specific IgE to egg white and to ovomucoid, and BAT to egg were significantly higher in this group compared with the other groups (*p* < 0.001).

### 3.2 The Mast Cell Activation Test to Egg Clearly Distinguishes Clinical Phenotypes of Egg Allergy

**FIGURE 2:**
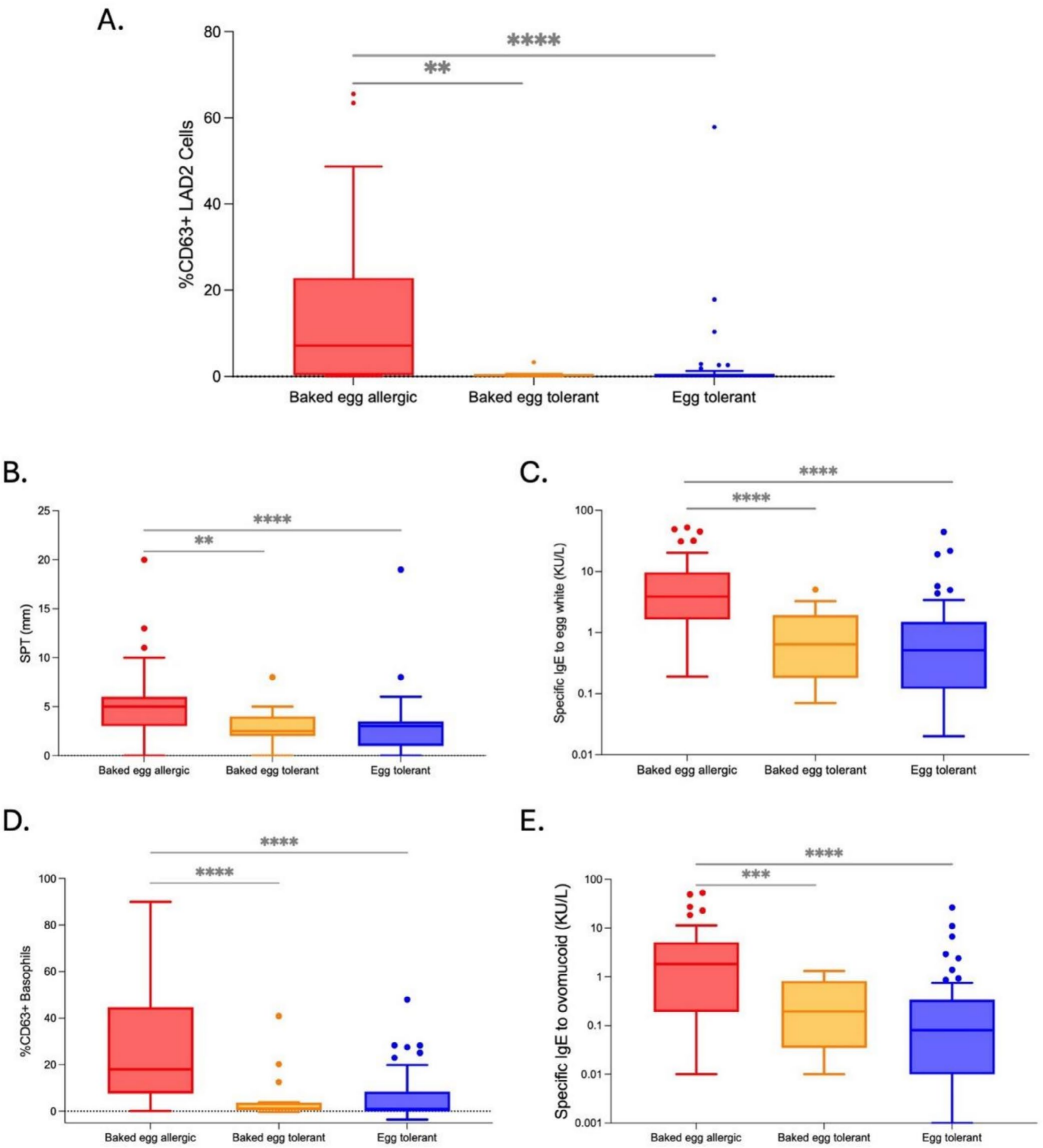
Results of mast cell activation to 1000 ng/mL of egg extract (A), skin prick test to egg extract (B), specific IgE to egg white (C), basophil activation to 100 ng/mL of egg extract (D) and specific IgE to ovomucoid (E) for the 3 subgroups of this cohort, that is, baked egg allergic (red, *n* = 58), baked egg tolerant (yellow, *n* = 16) and egg tolerant children (blue, *n* = 59). Comparison between groups is shown where statistically significant using Mann–Whitney *U* test—*p* values: ** < 0.01; *** < 0.001; **** < 0.0001.

Following sensitisation with plasma from patients with baked egg allergy, a greater proportion of LAD2 mast cells expressed the activation marker CD63, compared with that obtained with LAD2 cells sensitised with plasma from baked egg tolerant patients or patients tolerant to all forms of egg (Figure 2). The egg extract elicited greater mast cell activation than the baked egg preparation, evidencing the lower allergenicity of heated egg (Figure S3). The best discrimination between groups was obtained following mast cell stimulation with 1000 ng/mL of egg extract. This was also the optimal concentrations of egg extract to distinguish between allergic and tolerant groups to baked egg and loosely cooked egg in the MAT. Interestingly, patients with the intermediate egg allergy phenotype, able to tolerate baked egg but reacting to loosely cooked egg, had an intermediate degree of mast cell activation, that is, lower than that of baked egg allergic patients and higher than that of children who tolerate both baked and loosely cooked egg.

### 3.3 Clinical Utility of the Mast Cell Activation Test in Egg Allergy Diagnosis

Figure 2 illustrates the differences between results of MAT, BAT, SPT, specific IgE tests between the different phenotypes of egg allergy. Statistically significant differences were observed between baked egg allergic and baked egg tolerant and between baked egg allergic and all egg tolerant for all tests, but not between the children who reacted to loosely cooked egg whilst tolerating baked egg compared to the children who tolerated both baked and loosely cooked egg. This suggests that MAT and the other tests perform better to identify baked egg allergy than the other egg allergy phenotypes.

**FIGURE 3:**
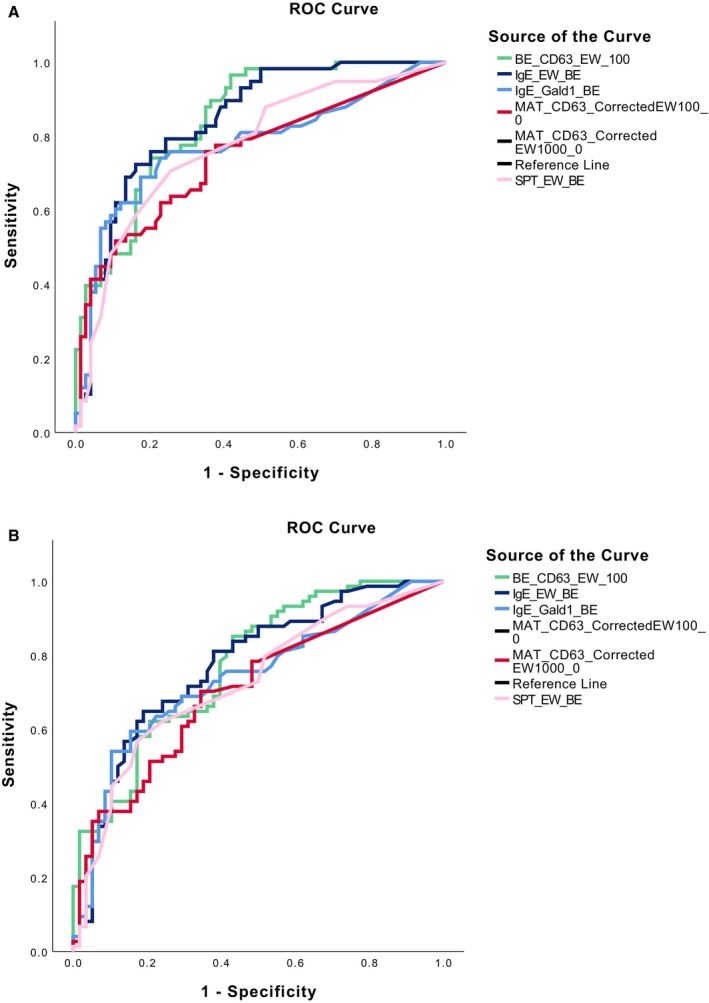
Receiver operator characteristic curve of the mast cell activation test to 1000 ng/mL to egg extract (MAT, in red) to distinguish allergic from tolerant children to baked egg (A) and loosely cooked egg (B) compared to skin prick test to egg extract (in pink), specific IgE to egg white (in dark blue), specific IgE to ovomucoid (in light blue) and the basophil activation test to 100 ng/mL of egg extract (in green).

In ROC curve analyses, MAT to egg demonstrated a good discriminative ability, with an area under the ROC curve (95% CI) of 0.749 (0.663; 0.835) for baked egg allergy and 0.704 (0.616; 0.793) for loosely cooked egg allergy (Figure 3). The optimal cut‐off for MAT to egg was 1.23% CD63+ LAD2 cells for baked egg allergy, with a 52% sensitivity and 89% specificity, 87% PPV and 58% NPV and 0.24% CD63+ LAD2 cells for loosely cooked egg allergy, with a 70% sensitivity and 66% specificity, 62% PPV and 73% NPV. Table 1 also shows maximal sensitivity and maximal specificity cut‐offs, which can be used to rule out and rule in for both baked egg and loosely cooked egg allergies, respectively, the diagnoses of baked egg and loosely cooked egg allergies.

**TABLE 1:** Diagnostic performance of optimal, maximal sensitivity and maximal specificity cut‐offs of the mast cell activation test to support the diagnosis of baked egg and loosely cooked egg allergies.

Allergy	Cut‐offs	%CD63+ LAD2 cells	Sensitivity	Specificity	Positive predictive value	Negative predictive value	Accuracy
Baked egg allergy	Maximal sensitivity	0.08	79%	55%	70%	68%	68%
	Optimal	1.23	52%	89%	87%	58%	68%
	Maximal specificity	57.72	2%	100%	100%	43%	44%
Loosely cooked egg allergy	Maximal sensitivity	0.04	78%	52%	57%	75%	64%
	Optimal	0.24	70%	66%	62%	73%	68%
	Maximal specificity	60.68	3%	100%	100%	56%	57%

Of note, the area under the ROC curve for MAT was lower than for other tests, such as SPT to egg, specific IgE to egg white and BAT to egg extract (0.775/0.845/0.846 and 0.726/0.773/0.774 for baked and loosely cooked egg allergies, respectively). As MAT is dependent on the presence of allergen‐specific IgE and efficient transfer of such IgE to the IgE receptors on the surface of mast cells, we compared the diagnostic performance of the tests considering patients with specific IgE to egg white greater than or equal to 1 KU/L (Table S5, Figure S4). The area under the ROC curve for MAT was higher than that of SPT but not of those of egg white‐specific IgE or BAT to egg. This poorer diagnostic performance of MAT is mainly due to a lower sensitivity, which is lower than desirable for a standalone test. MAT offers, however, advantages such as assessing the functional response of IgE (beyond levels) using stored samples collected in previous time‐points and not depending on fresh blood samples. BAT and MAT results were moderately correlated with one another (*R*s = 0.431, *p* < 0.001; Figure S5).

### 3.4 The Mast Cell Activation Test as a Tool to Assess Allergen‐Specific Antibody Function

In previous studies [12, 15], we used MAT to test the ability of IgE to induce effector cell response following allergen stimulation and the influence of different IgE characteristics on mast cell responses to the allergen. Herein, we compared the levels, specific activity, diversity and avidity of egg‐specific IgE and allergen‐specific IgG4/IgE ratios between allergic and tolerant children and related this with egg‐induced mast cell and basophil activation.

**FIGURE 4:**
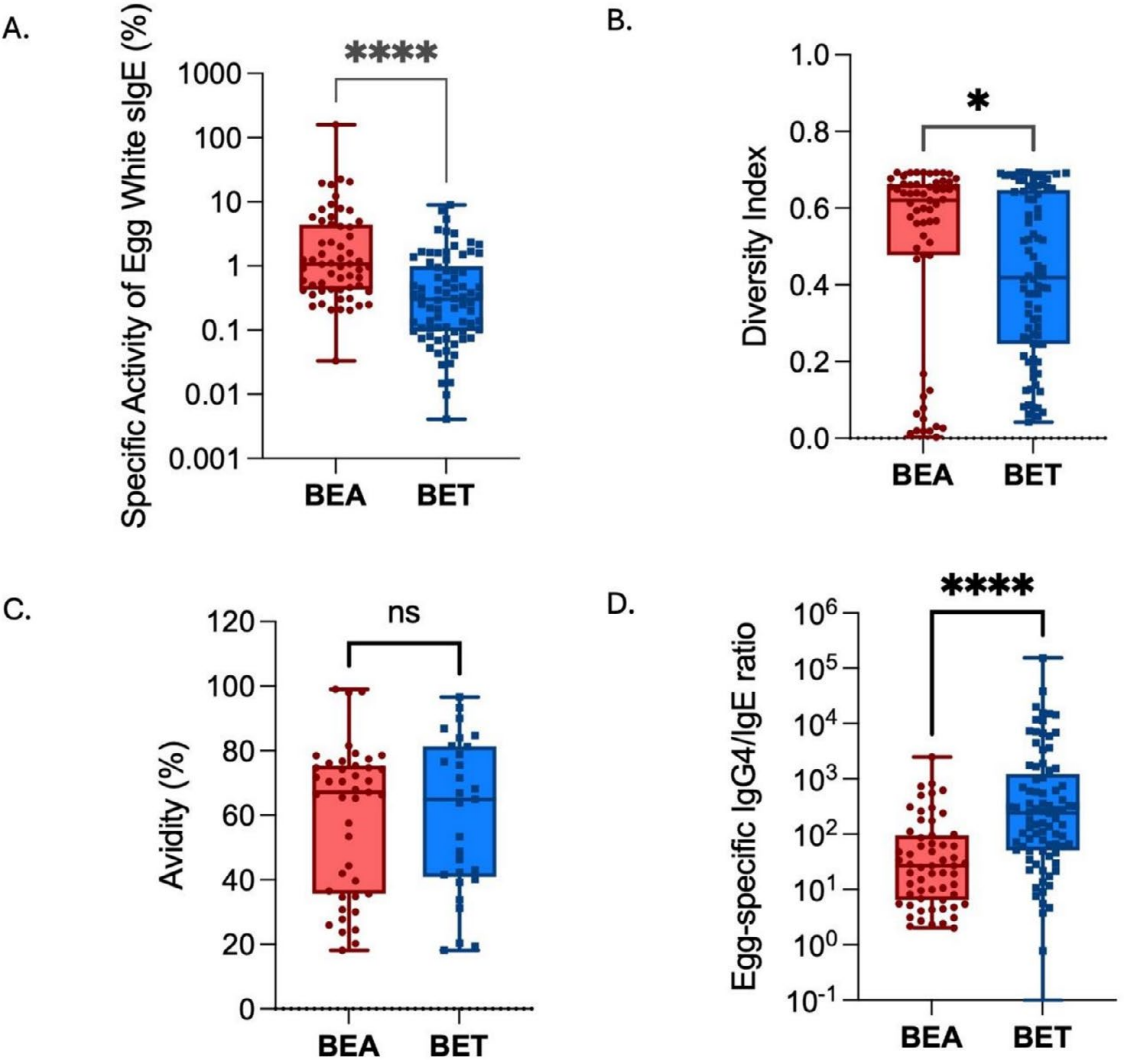
Functional characteristics of IgE to egg, including specific activity (A), diversity (B), avidity (C) and IgG4/IgE ratios (D) in samples from baked egg allergic (BEA) and baked egg tolerant (BET) children. Ns, non‐statistically significant. *p < 0.05; ****p < 0.0001.

**FIGURE 5:**
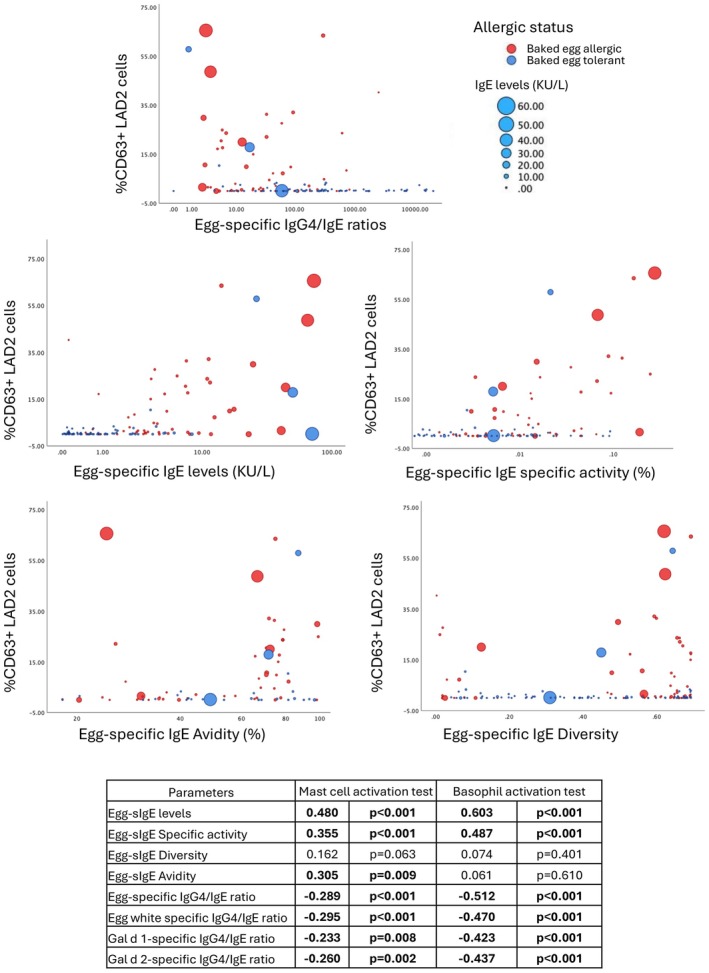
Correlation between egg‐specific IgE functional characteristics, IgG4/IgE ratios, basophil and mast cell activation to egg. Spearman correlation coefficients and *p* values are indicated (in bold when statistically significant).

Children reactive to all forms of egg had higher IgE specific activity and IgE diversity compared with IgE from baked egg tolerant children; however, there was no difference in the avidity of IgE for egg allergens between the two groups (Figure 4). IgE specific activity, specific IgE levels and IgE avidity for allergen were directly correlated with mast cell activation to egg (Figure 5). Similarly, IgE specific activity, specific IgE levels (but not IgE avidity for allergen) were directly correlated with basophil activation to egg (Figure S6). No correlation was observed between IgE diversity and MAT or BAT. Conversely, the ratio of IgG4 over IgE to egg, egg white, Gal d 1 and Gal d 2 was inversely correlated with both mast cell and basophil activation (Figure 5).

## 4 Discussion

Current tests do not accurately confirm diagnosis of egg allergy [21]. Cellular assays, like the BAT, have shown promise with reduced need for OFC compared to measuring allergen‐sIgE levels [11]. This is the first study of MAT in egg allergy, where we show, using samples from participants in the BAT2 egg study, that MAT can distinguish BE allergic and tolerant patients and is moderately correlated with BAT. However, overall, MAT does not have a better diagnostic performance than BAT or sIgE. Only when sIgE titres exceed 1 KU/L does MAT start to overcome the diagnostic accuracy of sIgE, with BAT remaining superior throughout. However, MAT has the advantage of not requiring fresh blood and can be used when BAT is not possible. MAT can also constitute a tool to measure the ability of IgE to convey the activator signal to mast cells following allergen stimulation and of IgG4 to interfere with IgE‐allergen interaction [12, 13]. BEA children had higher MAT and higher sIgE levels, specific activity and diversity (but not avidity) compared with BE tolerant. The direct correlation between these IgE characteristics (except diversity) and MAT, and the inverse correlation of egg‐specific IgG4/IgE ratios and MAT, indicate that functional characteristics of IgE are relevant to mast cell activation and that allergen‐sIgG4 can interfere with IgE‐mediated mast cell activation following allergen exposure, particularly in egg tolerance.

All patients included in this study underwent OFC, DBPCFC for all children aged 12 months or older, and thus were rigorously clinically phenotyped [11]. Blood samples were collected on the day of the OFC for most children, which contributes to the study rigour and the robustness of our findings. Clinical phenotype groups were defined based on the outcomes of the OFC, performed using food prepared in a standardised way by the research dietitian in the research kitchen, to baked egg and loosely cooked egg. Clinical phenotype groups did not include raw egg allergy, as this was not assessed, as children in the UK do not often consume egg in raw forms. For instance, in our clinic, we do not usually assess raw egg allergy, unless there has been a clear history of allergic reaction to raw egg or raw egg is part of the family's diet. There were 5 (3%) and 8 (9.6%) patients who did not have an outcome for OFC to baked egg and loosely cooked egg, respectively. However, this was a small proportion of patients and included a mix of likely allergic and likely non‐allergic patients; thus, their exclusion from analyses is unlikely to have biased the accuracy metrics of tests. Our cohort was formed of patients with suspected egg allergy whom we could not diagnose with certainty without doing an OFC; this enrichment with diagnostically ambiguous patients can potentially impair the performance of diagnostic tests but is a strength of our study, as this is precisely the group of patients who need additional and better tests. It may also be a contributing factor to the reduced proportion of children falling in the category of BE tolerant/LCE reactive compared with other cohorts. Additional factors that may have contributed to the attenuation of the difference between the BE and LCE challenge outcomes were the facts that our baked egg challenges reached a reasonably high dose of egg protein, that patients who passed the BE challenge consumed BE in the diet before attending the LCE challenge and that for some patients there was a longer than desired time gap between BE and LCE challenges [11].

The diagnostic performance of MAT was good and appropriate to support the diagnosis of egg allergy. Despite not performing better than the other tests (published previously in Ref. [11]), MAT has the advantage of using stored samples and allowing testing in the same experiment multiple samples collected at different time‐points. MAT is, however, dependent on the IgE levels, given that IgE from plasma needs to be transferred on to the mast cells and a standardised volume of plasma is used across patients. Samples with very low IgE levels may be unable to elicit cell activation, even if the mast cells are intrinsically able to respond. There are strategies to enhance the sensitivity of the assay by increasing the density of IgE receptor on the mast cell surface, such as adding IL‐4 to the culture, and increasing the ratio of volume of serum to cell suspension during sensitisation, which we adopted. However, to be established as a diagnostic assay, the MAT protocol needs to be standardised across patients, with the potential consequences in diagnostic performance for samples at both extremes of the IgE levels' distribution.

According to recent food allergy guidelines, cellular tests (namely BAT) are envisioned as a second step in the diagnostic process [22]. However, MAT was considered by the expert group driving the EAACI food allergy guidelines as requiring more research before it can be included as a recommended test for food allergy. In this study, the results of BAT and MAT were moderately correlated. Thus, MAT can potentially be used as a surrogate of BAT for food allergy diagnosis when fresh blood samples are not available. We and others [14, 23, 24] have previously reported on the utility of MAT to support the diagnosis of peanut allergy; herein, we report the utility of MAT to diagnose egg allergy. We confirm the utility of MAT to confirm the presence of egg allergy; however, the discriminative ability of MAT to egg was not superior to IgE to egg white, which questions the role of MAT in the diagnostic workflow for egg allergy. MAT had superior diagnostic performance compared to specific IgE when the titres exceeded the optimal IgE cut‐off of 2.25 KU/L. Therefore, MAT could be placed together with the other cellular assay BAT for patients for whom a fresh blood sample was not available to test on BAT or for patients with non‐responder basophils and egg white‐sIgE levels at the optimal cut‐off of 2.25 KU/L or above. Considering that the BAT2 study was enriched for children with an equivocal diagnosis after SPT and sIgE and about half of study participants had sIgE levels below 1 KU/L impacted on the overall performance of MAT to egg in this study. A better performance would be anticipated for a cohort with higher sIgE levels. FceRI expression, and therefore resulting IgE binding, can change after long periods in culture. As MAT is dependent on IgE capture by FceRI receptors on the surface of mast cells, FceRI needs to be monitored over time to ensure consistency and reproducibility of MAT results. In our experimental set up for MAT, FceRI expression and IgE binding levels are recorded in every experiment on both patient plasma‐sensitised cells and also non‐sensitised cells, as quality control checks.

MAT can also be useful when assessing the function of allergen‐specific antibodies and the interplay between allergen‐specific antibodies of different isotypes. We have previously used MAT to show that depletion of IgG4 from patient samples reduces the inhibitory effect on IgE‐mediated mast cell activation to peanut in peanut‐tolerant patients sensitised to peanut major allergens and in peanut allergic patients treated with peanut oral immunotherapy [13]. We have also shown that, after peanut oral immunotherapy, the ability of IgE to convey mast cell activation following peanut stimulation does not change, suggesting the clinical effect is driven by allergen‐specific blocking antibodies of different isotypes, such as IgG4 [19]. Interestingly, we have shown in this egg allergy study that the greater the IgG4/IgE ratio of egg‐specific antibodies the lower the mast cell and basophil activation, supporting the concept that blocking antibody effect is present in egg tolerance in IgE sensitised individuals and may result from the immune changes towards spontaneous resolution. This could be due to competition with IgE for allergen binding and/or by co‐engagement of IgE and IgG in the cell surface leading to inhibitory intracellular signalling via FcgRIIb. We previously [25] demonstrated that, although LAD2 cells express IgG receptors, IgG antibodies were not detected on their surface after sensitisation with serum and blocking FcγRIIα or FcγRIIβ did not modify mast cell activation in response to egg allergen stimulation, suggesting that extracellular competition is the main blocking mechanism of IgG, including IgG4.

The comparison between MAT and BAT as a tool to test IgE function and IgG4/IgE ratios is an interesting one. MAT is directly and moderately correlated with IgE characteristics, namely levels, proportion of IgE repertoire that is egg‐specific and avidity with diversity showing a trend towards statistical significance. The reason for the latter may be the fact that it only included two egg allergens, Gal d 1 and Gal d 2 and therefore may not be an optimal measure of IgE diversity. Ideally, IgE to more egg allergens would have been measured to better quantify the diversity of the egg‐specific IgE repertoire. BAT showed stronger correlations with egg‐specific IgE levels and specific activity but no correlation with diversity and avidity. The inverse correlation between cellular response and IgG4/IgE ratio was also stronger for BAT than MAT. Taken together, these comparisons suggest that MAT may be a good assessor of IgE function (although correlations were less strong than for BAT) but is unable to identify effector cell‐intrinsic differences in patients, both in terms of cell numbers, receptor expression levels and effector function. In contrast, BAT reflects better the interplay between IgE, allergen and blocking antibodies, possibly because it more closely resembles the physiology in vivo.

Our data suggests that egg‐specific IgE levels and specific activity are the dominant factors influencing effector cell response to allergen in egg allergy and that the changes with resolution seem to be more a change in quantity than in quality of IgE, in addition to the increase in IgG4/IgE ratios. One strength, but simultaneously a limitation of our study, is that patients are very similar clinically to the point of needing an OFC to determine whether they are allergic. Clearer differences between test results would likely be observed if the comparison was between highly egg allergic and completely tolerant patients. Further research is needed on the immune mechanisms underlying the changes in antibody levels and could inform strategies to modify the allergic immune response and induce tolerance.

In summary, MAT reflects the clinical phenotype of patients but is not as accurate as BAT for diagnosis, probably due to the more artificial nature of the assay, which uses cells that do not primarily belong to the patient being tested and have a fixed IgE receptor density that does not necessarily reflect the circulating IgE levels in the patient. MAT can be used as a surrogate of BAT when fresh blood is not available and BAT cannot be performed, and for mechanistic studies to explore antibody function and their immunomodulation using samples collected at different time‐points. Contrary to other mast cell assays, such as the Hoxb8 mast cell activation assay, the LAD2 cells are commercially available and this MAT can be established in other labs for diagnosis and for research, which is potentially valuable clinically and for reproducibility of findings, respectively.

## Author Contributions

V.H., V.C., N.R., Z.J., G.A. and M.K. performed laboratory analyses and conducted the statistical analysis. A.F.S., V.H., V.C. interpreted the data. A.F.S. conceived the study, designed the research, acquired the funding, supervised the project and drafted the first version of the manuscript. V.H. and V.C. critically revised the manuscript for important intellectual content. All authors approved the final manuscript.

## Funding

This work was supported by the Medical Research Council (MRC Fellowships MR/T032081/1 and MR/M008517/1, awarded to A.F. Santos), the UK National Institute for Health Research comprehensive Biomedical Research Centre award to Guy's and St Thomas' National Health Service (NHS) Foundation Trust, in partnership with the King's College London and King's College Hospital NHS Foundation Trust. Vikki Houghton was funded by King's College London as part of the ‘Neuro‐Immune Interactions in Health & Disease’ Wellcome Trust PhD Programme.

## Conflicts of Interest

Dr. Santos reports grants and personal fees from Medical Research Council (MR/M008517/1); grants from Asthma UK and the NIHR through the Biomedical Research Centre (BRC) award to Guy's and St Thomas' NHS Foundation Trust, during the conduct of the study; grants from Immune Tolerance Network/National Institute of Allergy and Infectious Diseases (NIAID, NIH), grants from Asthma UK; personal fees from Thermo Scientific, Novartis, Allergy Therapeutics, DBV, Sanofi‐Regeneron, RAPT Therapeutics, Nestle, IgGenix, Buhlmann, as well as research support from IgGenix, Buhlmann and Thermo Fisher Scientific through a collaboration agreement with King's College London. The other authors have nothing to disclose.

## Supporting information


**Table S1:** Dose regimens in grams of protein for baked egg challenges. *The initial doses will be given only in patients considered to be high‐risk (HR). **Cumulative dose does not include the High‐Risk doses.
**Table S2:** Dose regimens in grams of protein for DBPCFC to lightly cooked egg according to the different age groups.*The initial doses will be given only in patients considered to be high‐risk (HR). **Cumulative dose does not include the High Risk doses.
**Table S3:** Demographic and clinical features of the patients without outcome for oral food challenge to baked egg (*n* = 5) or loosely cooked egg (*n* = 8).
**Table S4:** Demographic and clinical features of the cohort tested on the mast cell activation test with clear clinical outcomes (*n* = 133). Statistically significant *p* values are indicated in bold (Kruskall‐Wallis test).
**Table S5:** Specific IgE and mast cell activation to egg in the subgroup of participants with specific IgE to egg white greater or equal to 1KU/L (*n* = 78), 2.25 KU/L (*n* = 56), 5 KU/L (*n* = 30) and 7 KU/L (*n* = 23). sIgE, specific IgE; N, number of participants; AUC ROC, area under the receiver operator characteristic curve; 95% CI, 95% confidence interval.
**Figure S1:** IgE avidity for egg measured with ImmunoCAP avidity assay using different concentrations of chaotropic agent sodium thyocianate (A) which disturbed IgE‐allergen interaction and reduced IgE binding (B), ****p < 0.0001 with Wilcoxon test.
**Figure S2:** Representative example of flow cytometry gating strategy of the mast cell activation test. (A) An example from a non‐egg‐allergic patient. In the first instance, gating is conducted on the negative control (top panel). L‐R: The gate in the first plot selects the LAD2 mast cell population, removing debris. Single cells are gated in the second plot, removing any duplicates. Live cells are selected in the third gate, using the viability dye under the pacific blue channel. The fourth plot thus allows quantification of CD63 expression (y axis, APC channel) within live cells (x axis, pacific blue channel). The top‐right quadrant of the contour plot represents CD63+ cells. Position of the y‐axis quadrant is determined and is maintained constant for all other analyses, as demonstrated with egg white extract 1000 ng/mL (middle panel) and the positive control (anti‐immunoglobulin‐E, 1 μg/mL). (B) An example from an allergic patient, where increased % CD63 can be seen in the egg extract‐stimulated condition (middle panel, far right plot) compared to the non‐stimulated control (top panel). For all plots, the text below each plot refers to the parent population, and the total cell numbers in that population. The text within the top‐right of each plot refers to the output population and the percentage of cells gated within it.
**Figure S3:** Mast cell activation following stimulation with egg extract and baked egg using LAD2 cells sensitised with plasma from baked egg allergic (*n* = 35), baked egg tolerant (*n* = 13) and egg tolerant children (*n* = 36) for whom both egg allergen extract and baked egg were tested in a dose–response.
**Figure S4:** Receiver operator characteristic curve of the mast cell activation test to 1000 ng/mL to egg extract (MAT, in red), and specific IgE to egg (in blue) to distinguish allergic from tolerant children to baked egg among children with specific IgE to egg white greater or equal to (A) 1 KU/L; (B) 2.25 KU/L.
**Figure S5:** Correlation between mast cell activation to egg and basophil activation to egg. Spearman correlation coefficient was *R*s = 0.431, *p* < 0.001.
**Figure S6:** Correlation between egg‐specific IgE functional characteristics, IgG4/IgE ratios and basophil activation to egg.

## Data Availability

The data that support the findings of this study are available on request from the corresponding author. The data are not publicly available due to privacy or ethical restrictions.
