# Supplementary material for: IgE Antibody Function and Mast Cell Activation to Egg in Children Undergoing Baked and Loosely Cooked Egg Challenges
Source: Allergy. 2026 Apr 29;81(8):2874–84. doi: 10.1111/all.70368 (PMC13466162; doi:10.1111/all.70368)
Supplement: Supplementary file 1 [file ALL-81-2874-s001.docx]

**Online E-Methods**

**Food preparation for double-blind placebo-controlled food challenges**

The blinded doses of baked egg and loosely cooked egg were prepared by the clinical research staff according to a standardised food preparation protocol developed by the study's Registered Dietitian. For baked egg, biscuits were baked in large batches using a standardised protocol, frozen at -20C until use and defrosted on the day of the challenge. Large sized eggs were used for the food challenges to ensure consistency with the protocol cooking times. The blinded doses consisted of boiled egg cooked until the egg white and egg yolk was set for a standardised time of 8:30 minutes at 900W in the study microwave (Samsung MK700K Convection Microwave Oven). Following cooking, the eggs were placed into cold water to reduce the heat and thus cooking, before shelling, blending and combining with a food vehicle for blinding and weighing as per the study food preparation protocol. The open doses consisted of scrambled, whole egg cooked until the egg was just set, for a standardised time of 1:45 minutes at 450W in the study microwave (as above).

**Table E1.** Dose regimens in grams of protein for baked egg challenges. *The initial doses will be given only in patients considered to be high-risk (HR). **Cumulative dose does not include the High-Risk doses

| Age | | 6-12 months | 1-3 years | 4-10 years | 11-16 years |
| --- | --- | --- | --- | --- | --- |
| Type of challenge | | Open | DBPCFC | DBPCFC | DBPCFC |
| Number of placebo doses randomly interspersed | | 0 | 1 | 1 | 1 |
| Active doses  (g) | High Risk Dose 1* | 0.003 | 0.003 | 0.003 | 0.003 |
|  | High Risk Dose 2* | 0.01 | 0.01 | 0.01 | 0.01 |
|  | Dose 1 | 0.03 | 0.03 | 0.03 | 0.03 |
|  | Dose 2 | 0.1 | 0.1 | 0.1 | 0.1 |
|  | Dose 3 | 0.2 | 0.3 | 0.3 | 0.3 |
|  | Dose 4 | 0.4 | 0.6 | 0.6 | 0.6 |
|  | Open dose | 0.8 | 1.0 | 1.5 | 2.5 |
|  | Cumulative protein dose** | 1.53 | 2.03 | 2.53 | 3.53 |
|  | Total number of muffin/cookies | 1.0 | 1.3 | 1.6 | 2.2 |

**Table E2.** Dose regimens in grams of protein for DBPCFC to lightly cooked egg according to the different age groups.*The initial doses will be given only in patients considered to be high-risk (HR). **Cumulative dose does not include the High Risk doses.

| Age | | 6-12 months | 1-3 years | 4-10 years | 11-16 years |
| --- | --- | --- | --- | --- | --- |
| Food | | LIGHTLY COOKED EGG | | | |
| Type of challenge | | Open | DBPCFC | DBPCFC | DBPCFC |
| Number of placebo doses randomly interspersed | | 0 | 1 | 1 | 1 |
| Active doses  (g) | High Risk Dose 1* | 0.003 | 0.003 | 0.003 | 0.003 |
|  | High Risk Dose 2* | 0.01 | 0.01 | 0.01 | 0.01 |
|  | Dose 1 | 0.03 | 0.03 | 0.03 | 0.03 |
|  | Dose 2 | 0.1 | 0.1 | 0.1 | 0.1 |
|  | Dose 3 | 0.3 | 0.3 | 0.3 | 0.3 |
|  | Dose 4 | 1.0 | 1.0 | 1.0 | 1.0 |
|  | Open dose 1 | 2.0 | 2.0 | 2.0 | 2.0 |
|  | Open dose 2 | 3.0 | 4.0 | 5.0 | 6.0 |
|  | Cumulative protein dose** | 6.43 | 7.43 | 8.43 | 9.43 |
|  | Total number of 50g medium eggs | 0.9 | 1.0 | 1.2 | 1.3 |

**Table E3.** Demographic and clinical features of the patients without outcome for oral food challenge to baked egg (n=5) or loosely cooked egg (n=8).

| Food | Age | Gender | History of reaction to egg | SPT egg white (mm) | Specific IgE to egg white (KU/L) | BAT to egg (%CD63+ basophils) | MAT to egg (%CD63+ LAD2 cells) | OFC |
| --- | --- | --- | --- | --- | --- | --- | --- | --- |
| Baked Egg | 12 Y | M | No | 14 | 2.89 | 34.19% | 3.86% | Refusal to eat the first dose |
|  | 4 Y | F | Yes | 4 | 7.09 | 26.52% | 16.49% | Could not consume target cumulative dose in full |
|  | 3 Y | M | Yes | 3 | 4.90 | 9.10% | 13.52% | Could not consume target cumulative dose in full |
|  | 13 Y | F | Yes | 3 | 2.42 | 0.87% | 0.25% | Subjective symptoms (Abdominal pain 4/10) |
|  | 1 Y | M | Yes | 5 | 0.14 | 4.24% | 0.84% | Could not consume target cumulative dose in full |
| Cooked egg | 2 Y | M | No | 3 | 4.77 | 2.94% | 28.80% | OFC BM negative,  did not attend OFC |
|  | 5 Y | F | Yes | 2 | 6.96 | 31.51% | 6.70% |  |
|  | 1 Y | F | Yes | 3 | 0.07 | 0.99% | 0.00% |  |
|  | 7 Y | F | Yes | 4 | 1.27 | 32.58% | 0.08% |  |
|  | 2 Y | F | Yes | 6 | 0.30 | 7.07% | 1.01% |  |
|  | 2 Y | F | Yes | 2 | 0.27 | 0% | 0.00% |  |
|  | 2 Y | F | Yes | 2 | 0.25 | 0.29% | 0.86% |  |
|  | 9 Y | M | Yes | 0 | 0.18 | 0% | 0.06% |  |

**Table E4.** Demographic and clinical features of the cohort tested on the mast cell activation test with clear clinical outcomes (n=133). Statistically significant p values are indicated in bold (Kruskall-Wallis test).

| **Characteristics** | **Baked egg allergic**  **(n=58)** | **Baked egg tolerant**  **(n=16)** | **Egg tolerant**  **(n=59)** | **P value** |
| --- | --- | --- | --- | --- |
| Age (years) | 6.2 (3.2; 8.9) | 4.9 (2.5; 7.0) | 4.9 (2.3; 9.8) | 0.561 |
| Gender (male/female) | 33 (57%)/25 (43%) | 8 (50%) / 8 (50%) | 35 (59%)/24 (42%) | 0.799 |
| Ethnicity   - White | 38 (66%) | 7 (44%) | 37 (63%) |  |
| - Black | 6 (10%) | 1 (6%) | 5 (9%) |  |
| - Asian | 4 (7%) | 2 (13%) | 5 (9%) | 0.329 |
| - Chinese | 3 (5%) | 0 (0%) | 0 (0%) |  |
| - Mixed | 4 (7%) | 5 (31%) | 9 (15%) |  |
| - Other | 3 (5%) | 1 (6%) | 3 (5%) |  |
| Eczema – n (%) | 46 (79%) | 13 (81%) | 49 (83%) | 0.875 |
| Rhinitis – n (%) | 25 (43%) | 6 (38%) | 21 (37%) | 0.700 |
| Asthma – n (%) | 23 (40%) | 1 (6%) | 16 (27%) | **0.029** |
| Other food allergies – n (%) | 45 (78%) | 10 (63%) | 37 (63%) | 0.181 |
| Skin prick test to egg extract (mm) | 5 (3; 6) | 2 (2; 4) | 3 (1; 4) | **<0.001** |
| Specific IgE to egg (KU/L) | 3.85 (1.80; 9.80) | 0.62 (0.20; 2.06) | 0.58 (0.13; 1.50) | **<0.001** |
| Specific IgE to egg white (KU/L) | 3.87 (1.87; 9.73) | 0.64 (0.18; 1.93) | 0.56 (0.12; 1.54) | **<0.001** |
| Specific IgE to ovalbumin (KU/L) | 2.03 (0.95; 5.45) | 0.43 (0.11; 1.29) | 0.50 (0.14; 0.97) | **<0.001** |
| Specific IgE to ovomucoid (KU/L) | 1.87 (0.30; 5.13) | 0.20 (0.04; 0.82) | 0.10 (0.01; 0.35) | **<0.001** |
| BAT to egg extract (%CD63+ Basophils) | 18.36 (8.31; 45.09) | 1.22 (0.43; 3.69) | 1.19 (0; 8.76) | **<0.001** |
| MAT to egg extract (%CD63+ LAD2 cells) | 1.81 (0.17; 18.25) | 0.27 (0.08; 0.53) | 0.01 (0; 0.65) | **<0.001** |

**Table E5.** Specific IgE and mast cell activation to egg in the subgroup of participants with specific IgE to egg white greater or equal to 1KU/L (n=78), 2.25 KU/L (n=56), 5 KU/L (n=30) and 7 KU/L (n=23). sIgE, specific IgE; N, number of participants; AUC ROC, area under the receiver operator characteristic curve; 95%CI, 95% confidence interval.

| **Egg white sIgE >=** | **N** | **Test** | **AUC ROC (95% CI)** |
| --- | --- | --- | --- |
| 1 KU/L | 78  (48 BEA, 30 BET) | sIgE | 0.695 (0.569; 0.821) |
|  |  | MAT | 0.709 (0.591; 0.827) |
| 2.25 KU/L | 56  (42 BEA, 14 BET) | sIgE | 0.530 (0.347; 0.712) |
|  |  | MAT | 0.636 (0.461; 0.811) |
| 5 KU/L | 30  (24 BEA, 6 BET) | sIgE | 0.417 (0.125; 0.709) |
|  |  | MAT | 0.642 (0.383; 0.902) |
| 7 KU/L | 23  (20 BEA, 3 BET) | sIgE | 0.117 (0; 0.259) |
|  |  | MAT | 0.483 (0.108; 0.859) |

**Figure E1.** IgE avidity for egg measured with ImmunoCAP avidity assay using different concentrations of chaotropic agent sodium thyocianate (A) which disturbed IgE-allergen interaction and reduced IgE binding (B), ****p<0.0001 with Wilcoxon test.

A.	B.

**Figure E2.** Representative example of flow cytometry gating strategy of the mast cell activation test. **(A)** An example from a non-egg-allergic patient. In the first instance, gating is conducted on the negative control (top panel). L-R: The gate in the first plot selects the LAD2 mast cell population, removing debris. Single cells are gated in the second plot, removing any duplicates. Live cells are selected in the third gate, using the viability dye under the pacific blue channel. The fourth plot thus allows quantification of CD63 expression (y axis, APC channel) within live cells (x axis, pacific blue channel). The top-right quadrant of the contour plot represents CD63+ cells. Position of the y-axis quadrant is determined and is maintained constant for all other analyses, as demonstrated with egg white extract 1,000 ng/ml (middle panel) and the positive control (anti-immunoglobulin-E, 1 μg/ml). **(B)** An example from an allergic patient, where increased % CD63 can be seen in the egg extract-stimulated condition (middle panel, far right plot) compared to the non-stimulated control (top panel). For all plots, the text below each plot refers to the parent population, and the total cell numbers in that population. The text within the top-right of each plot refers to the output population and the percentage of cells gated within it.

**Figure E3.** Mast cell activation following stimulation with egg extract and baked egg using LAD2 cells sensitized with plasma from baked egg allergic (n=35), baked egg tolerant (n=13) and egg tolerant children (n=36) for whom both egg allergen extract and baked egg were tested in a dose-response.

**Figure E4.** Receiver operator characteristic curve of the mast cell activation test to 1000ng/ml to egg extract (MAT, in red), and specific IgE to egg (in blue) to distinguish allergic from tolerant children to baked egg among children with specific IgE to egg white greater or equal to A. 1 KU/L; B. 2.25 KU/L.

***A.***

**B.**

**Figure E5.** Correlation between mast cell activation to egg and basophil activation to egg. Spearman correlation coefficient was Rs=0.431, p<0.001.

**Figure E6.** Correlation between egg-specific IgE functional characteristics, IgG4/IgE ratios, and basophil activation to egg.
